# ERCC4: a potential regulatory factor in inflammatory bowel disease and inflammation-associated colorectal cancer

**DOI:** 10.3389/fendo.2024.1348216

**Published:** 2024-03-07

**Authors:** Runjie Shi, Shanping Wang, Ying Jiang, Guoqiang Zhong, Mingsong Li, Yan Sun

**Affiliations:** Department of Gastroenterology, Guangdong Provincial Key Laboratory of Major Obstetric Diseases, Guangdong Provincial Clinical Research Center for Obstetrics and Gynecology, The Third Affiliated Hospital of Guangzhou Medical University, Guangzhou, China

**Keywords:** excision repair cross complementation group 4, inflammatory bowel disease, colorectal cancer, colitis-associated cancer, NER (nucleotide excision repair)

## Abstract

The pathogenesis of inflammatory bowel disease (IBD) remains unclear and is associated with an increased risk of developing colitis-associated cancer (CAC). Under sustained inflammatory stimulation in the intestines, loss of early DNA damage response genes can lead to tumor formation. Many proteins are involved in the pathways of DNA damage response and play critical roles in protecting genes from various potential damages that DNA may undergo. ERCC4 is a structure-specific endonuclease that participates in the nucleotide excision repair (NER) pathway. The catalytic site of ERCC4 determines the activity of NER and is an indispensable gene in the NER pathway. ERCC4 may be involved in the imbalanced process of DNA damage and repair in IBD-related inflammation and CAC. This article primarily reviews the function of ERCC4 in the DNA repair pathway and discusses its potential role in the processes of IBD-related inflammation and carcinogenesis. Finally, we explore how this knowledge may open novel avenues for the treatment of IBD and IBD-related cancer.

## 1 Introduction

Colorectal cancer (CRC) is one of the most common malignancies, ranking third globally in terms of incidence and second in mortality among malignant tumors, and is the leading digestive system malignancy in terms of both incidence and morbidity (1, 2). In recent years, the incidence of CRC in China has been increasing year by year, now ranking second in digestive system incidence and first in morbidity of malignant tumors (3–5). In 1863, the German pathologist Rudolf Virchow first proposed the idea that “tumors originate from chronic inflammation (6), and previous studies have shown that “tumor-related inflammation is the seventh major characteristic of tumors”. According to recent epidemiological investigations, a variety of tumors are related to inflammation, and chronic inflammation of the intestinal mucosa is an important high-risk factor for inducing colorectal cancer (7, 8).

Inflammatory bowel disease (IBD), including ulcerative colitis (UC) and Crohn’s disease (CD), is a chronic, non-specific, and recurrent disease that can affect the entire digestive tract. IBD is an autoimmune disorder that presents as immune hyperactivity (9–11). As a chronic disease, various intestinal or extraintestinal complications and/or complications are easy to occur throughout the course of IBD, including bowel fistula, gastrointestinal bleeding, and tumors (12). While CD and UC have different clinical symptoms, they both have characteristics of chronic inflammation and immune response dysregulation. Colon cancer induced by inflammation is the most serious complication of IBD. Based on its pathological mechanisms, clinical characteristics, and pathological types, colorectal cancer transformed from colitis is collectively referred to as colitis-associated cancer (CAC) (13, 14). Studies have shown that colorectal cancer is the leading cause of death in IBD patients, accounting for 10%-15% of all-cause mortality in IBD patients. The risk of IBD patients developing CRC is about three times that of the general population, and with the expansion of the IBD patient population, the detected population of IBD-related CRC is also increasing (15–17). IBD patients face twice the risk of developing CRC compared to the general population, accounting for 1-2% of all CRC cases. A meta-analysis examining UC-associated neoplasia suggests an overall cancer incidence of 3.7% among IBD patients. Furthermore, the risk of CAC escalates annually with disease duration, with reported incidence rates of 2% within 10 years of IBD diagnosis, 8% after 20 years, and 18% after 30 years (18, 19).

Compared with the pathogenesis of sporadic CRC, there are some obvious differences in the pathogenesis of CAC. The process of developing sporadic CRC from adenoma to cancer involves early loss of tumor suppressor gene APC and activation of the typical Wnt/β-catenin pathway, accompanied by mutation of the oncogene K-ras, and ultimately, loss of the tumor suppressor gene p53 (20–22). With the development of multi-omics analysis and the construction of chronic gastrointestinal inflammatory animal models, more and more evidence suggests that intestinal inflammation caused by IBD can induce genetic changes closely related to the development of CAC, oxidative stress-driven DNA damage, early loss of p53, and host immune response dysregulation. The development of CAC follows a complex trajectory, during which DNA damage and repair are consistently involved (23, 24).

This review provides the current research and data on the function of ERCC4, emphasizing its potential therapeutic role in IBD and associated cancer. This study will contribute to a better understanding of the mechanisms underlying the development and progression of IBD and provides crucial information for the development of related treatment strategies.

## 2 IBD and the development of inflammation-associated colorectal cancer involve DNA damage

DNA damage poses a serious threat to the health of the body, including DNA mismatches during replication and DNA damage caused by endogenous reactive oxygen species (ROS). Additionally, various physical and chemical substances from the external environment, such as UV radiation and carcinogenic chemicals, can also attack DNA. Therefore, cells have evolved a DNA damage response (DDR) to counteract DNA damage (25, 26). The DDR allows cells to survive in the face of genomic instability or leads to senescence or programmed cell death of irreparable damaged cells. If DNA damage cannot be repaired or is repaired incorrectly, it may be transmitted to daughter cells during cell division, accumulate over time, and eventually lead to mutations in important genes (such as activation of oncogenes and inactivation of tumor suppressor genes), ultimately resulting in the development of cancer (27, 28). Somatic mutations are an important mechanism for gene inactivation at the DNA sequence level. Somatic mutations correlated with DDR pathways are found in some hereditary cancer syndromes, such as hereditary nonpolyposis colorectal cancer (Lynch syndrome), as well as hereditary breast and ovarian cancer (29–31). Besides these inherited syndromes, DDR-related germ-line mutations have been identified in sporadic cancers as well. DDR-related genes can also undergo high-frequency somatic mutations. In a pan-cancer analysis of 17 tumor types, 72 key DDR genes were found to harbor somatic mutations, with at least 1% of samples in a given tumor having mutations, and appearing in more than one type of cancer (32). Research by Chen R found that the level of DNA damage in UC colon cells is higher compared to the normal control group, especially in UC patients with dysplasia and cancer (33).

## 3 The ERCC4 gene and its biological functions

As mentioned above, both endogenous and exogenous carcinogens can cause DNA damage, and the body can repair damaged DNA through the process of DNA damage repair, which helps maintain genomic stability and suppress the development of cancer (26). Different DNA repair mechanisms have evolved in response to various types of damage to prevent the accumulation of DNA defects and preserve genetic information. The DDR system is broadly categorized into three major classes. The first class is ubiquitous across prokaryotes and eukaryotes and includes mechanisms such as direct repair, base excision repair (BER), and mismatch repair (MMR) (34). The second class is specific to eukaryotes and primarily evolved to counteract single and double-strand breaks in DNA, exemplified by homologous recombination (HR) and non-homologous end joining (NHEJ). The third class encompasses nucleotide excision repair (NER), which has evolved to combat DNA damage leading to distortion of the DNA helical structure, such as UV-induced lesions or chemical cross-linking (35). NER is the most important pathway among these repair pathways and can repair cellular damage caused by DNA double helix distortion (36–39).

Excision repair cross complementation group 4 (ERCC4), also known as XPF, is located on human chromosome 16p13.12 and consists of 11 exons, spanning approximately 28.2 kb. ERCC4 is widely expressed in various normal tissues in mammals, including the skin, nervous system, reproductive system, endothelial cells, and various immune cells including T lymphocytes and macrophages (40). The protein encoded by ERCC4 is one of the key enzymes in the NER pathway and is involved in the formation of nucleases, DNA repair, and maintenance of chromosomal stability. The ERCC1-ERCC4 complex is a dual-subunit structure-specific endonuclease. This endonuclease specifically cleaves DNA near the junction between single-stranded and double-stranded DNA, excising damaged 5’ ends and linking newly synthesized DNA strands to the intact portion. ERCC1-ERCC4 plays a central role in NER. Furthermore, ERCC1-ERCC4 contributes to several other DNA repair pathways, such as repairing double-strand breaks (DSBs) and interstrand crosslinks (ICLs), possibly due to its unique catalytic cleavage properties (41, 42).

**Figure 1:**
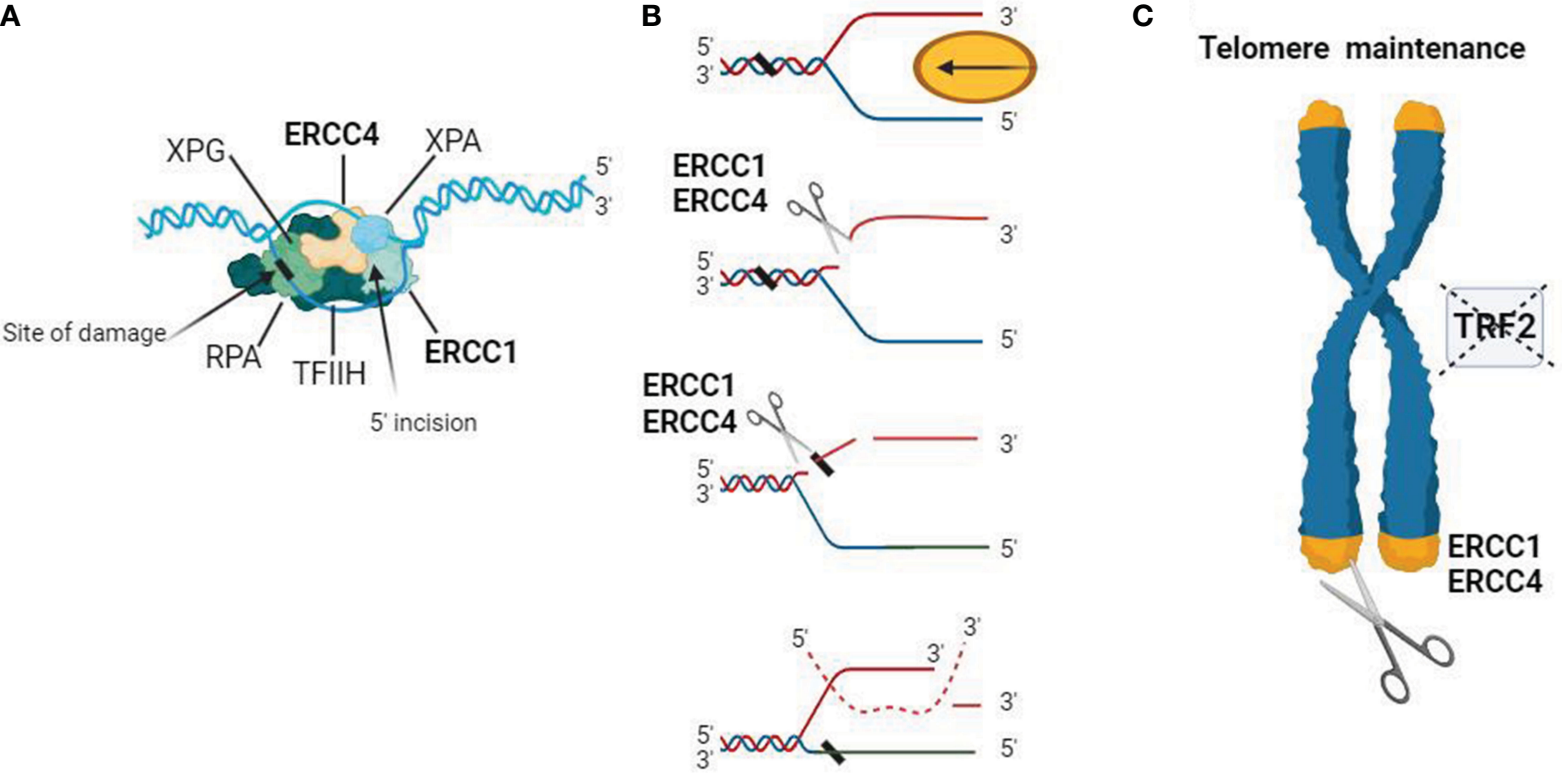
Schematic representation of the biological functions of the ERCC4/XPF complex. **(A)** DNA substrate example for the ERCC1-ERCC4 endonuclease. During NER, the ERCC1-ERCC4 endonuclease introduces a heteroduplex 5′ incision on one strand. The pre-incision complex is shown here, composed of the transcription factor IIH (TFIIH), XPA, RPA, and XPG. The DNA strand is separated in an ATP-dependent reaction to create a substrate for ERCC1-ERCC4 cleavage. The ERCC1/ERCC4 complex is recruited to the site of damage, and the subsequent 3′ incision causes a conformational change in XPG, which catalyzes the 5′ incision around the lesion. The cleaved strand dissociates from the junction in a 5′ to 3′ direction. **(B)** Interstrand crosslink repair model. Due to replication stalling or with the help of a DNA helicase, damaged DNA forms a Y-structure near the crosslink site. ERCC1/ERCC4 first cleaves on the 5′ side of one arm of the crosslink and then makes an additional incision on the 3′ side to complete the repair. Dashed lines represent the invading homologous DNA strand. **(C)** ERCC1/ERCC4 plays a role in telomere maintenance, where it degrades overhangs containing 3′G when the function of telomere-binding protein 2 (TRF2) is inhibited.

Studies have shown that ERCC4 often functions as a complex with ERCC1 in NER, and it is involved in double-strand break repair, telomere maintenance, and immunoglobulin switching (**Figure 1**). Both ERCC1 and ERCC4 are indispensable for the function of the ERCC1-ERCC4 complex in NER, although the actual catalytic region of this complex resides in the ERCC4 protein (43–45).

### 3.1 The role of ERCC4 in NER

(GG-NER) and transcription-coupled NER (TC-NER) (46). XPC-RAD23B initiates GG-NER by recognizing DNA distortion, followed by the assembly of TFIIH, XPA, RPA, XPG, and ERCC1-ERCC4 to form a pre-incision complex. ERCC1-ERCC4, a key endonuclease, interacts significantly with XPA during repair in both replicating and non-replicating cells. TC-NER is triggered by RNA polymerase II stalling, recruiting repair proteins like CSA, CSB, TFIIH, XPA, and RPA to the damaged site. ERCC1-ERCC4 and XPG make incisions on the 5’ and 3’ sides of the lesioned DNA strand, releasing fragments approximately 27-29 nucleotides in length (47, 48).

Inherited mutations in NER-related genes are associated with UV sensitivity and cancer susceptibility. NER deficiencies increase sensitivity to platinum-based chemotherapy due to impaired interstrand crosslink repair. Monitoring key NER enzyme levels can aid in patient stratification. Despite potential downregulation effects of cyclosporine and cetuximab on XPG and ERCC1-ERCC4 expression, small molecule NER inhibitors remain challenging to develop (49).

### 3.2 The role of ERCC4 in DSB

DSB can arise from external sources like ionizing radiation and chemical agents, as well as internal factors during DNA replication, recombination, and cellular metabolism. They are among the most deleterious DNA damages and necessitate repair for genome stability (50). Cells lacking ERCC1-ERCC4 exhibit heightened sensitivity to DSB-inducing agents, such as X-rays and gamma radiation, compared to normal cells. ERCC1-ERCC4 deficiency in mice and mouse fibroblasts leads to increased γH2AX foci (DNA damage markers) and chromosomal abnormalities post-ionizing radiation exposure. DSB repair primarily occurs through non-homologous end joining (NHEJ) and homologous recombination (HR) (51, 52). The ERCC1/ERCC4 endonuclease plays a crucial role in efficient single-strand annealing and gene conversion in mammalian cells. Specifically, during single-strand annealing, ERCC1-ERCC4 performs DNA incision. Moreover, ERCC4 interacts with Rad52’s N-terminal DNA binding region independently of DNA, aiding ERCC1-ERCC4 in cleaving 3’-overhangs and processing recombination intermediates during DSB repair. In mammalian cells, ERCC1-ERCC4 is vital for removing long non-homologous ends targeted for homologous recombination. ERCC1-ERCC4 may also engage in NHEJ when substantial 3’-end trimming is necessary at DSB ends, regulated by DNA-dependent protein kinase (DNA-PK) within the NHEJ complex (51–53). These observations emphasize the pivotal role of ERCC1-ERCC4 in DSB repair pathways.

Research has found an increase in DSBs levels in the DNA of IBD patients (54, 55). Wiebke Lessel et al. discovered that histone H2AXγ could serve as a biomarker for UC-associated colorectal cancer, used to evaluate DNA damage induced by ROS in UC patients. The mechanism of its formation remains unclear, possibly involving changes in colonic epithelial cell apoptosis or cell division (56). Whether ERCC4 affects the development process of diseases in IBD and IBD-related CAC through regulating the DSB pathway is not fully understood at present, and further research is anticipated.

### 3.3 The role of ERCC4 in BER

Organisms constantly undergo subtle genomic changes due to endogenous genotoxins like reactive ROS, ionizing radiation, and environmental alkylating agents. BER pathway primarily handles minor DNA alterations such as single-strand breaks (SSBs) (57). Initiated by damaged bases, BER excises and replaces them with newly synthesized DNA. Apurinic/apyrimidinic endonuclease (APE) cleaves at the AP site, creating a 3′OH end at the damage site. DNA polymerase and ligase then seal the gap left by the damaged base removal. In budding yeast, the absence of AP endonuclease doesn’t hinder the processing of the 3’ end, relying on Rad1-Rad10. Similarly, mammalian ERCC1-ERCC4 can remove 3’-phosphoglycolate from DNA’s 3’ end (resulting from oxidative attack) *in vitro*. Hence, ERCC1-ERCC4 may act as a backup for processing oxidatively damaged DNA in mammalian cells (57–59).

### 3.4 The role of ERCC4 in MMR

MMR is a conservative mechanism addressing mispaired nucleotides stemming from DNA damage or replication errors. Despite the proofreading function of replicative polymerases, some nucleotides often elude this process, leading to mismatches. MMR encompasses mismatch recognition, nascent strand incision, mismatch excision, and repair synthesis. In higher organisms, MutSα (MSH2-MSH6 heterodimer) or MutSβ (MSH2-MSH3 heterodimer) identifies mismatches (60).

Human SLX4 interacts with MSH2 and MSH3, forming MutSβ, crucial for repairing heteroduplex loops in DNA replication. However, the interaction between SLX4 and MutSβ, and their role in genome stability, was poorly understood until recently. MutSβ is also involved in removing 3’-nonhomologous tails during single-strand annealing (SSA), vital for repairing DSBs between direct repeats (60, 61). Repair involves annealing repeats on both sides of the DSB, resulting in the loss of intervening sequences, dependent on RAD52. MutSβ is pivotal for removing nonhomologous tails generated during annealing reactions. For instance, in budding yeast, MutS is recruited to DSB sites via Rad52. MutSβ aids in tail removal by interacting with the yeast homolog of ERCC4-ERCC1 (Rad1-Rad10) and recruiting them (60–62).

### 3.5 The role of ERCC4 in ICLs

Addressing DNA ICLs presents significant challenges due to their interference with DNA replication and transcription. Cells lacking ERCC1 and ERCC4 display heightened sensitivity to ICL-inducing agents like cisplatin. Conversely, deficiencies in other NER genes result in relatively lower sensitivity to ICL-inducing agents, underscoring the pivotal role of ERCC1-ERCC4 in ICL repair (63). Mutations in ERCC4 and ERCC1 have been linked to impairments in the incision or ‘uncoupling’ stage of ICL repair. ERCC1-ERCC4 enhances nuclease activity during ICL repair through interactions with SLX4 and ubiquitinated FANCD2. A subset of ERCC4-ERCC1 interacts with SLX1-SLX4 in human cells, facilitated by direct interaction between ERCC4 and the MEI9 interaction region (MLR) of SLX4. SLX4 promotes ERCC4-mediated cleavage of branched DNA structures, including replication forks and ICLs, consistent with its role in ICL repair. Additionally, SLX4IP is indispensable for efficient ICL repair by interacting with SLX4 and ERCC4-ERCC1. These findings underscore the importance of the SLX4-SLX4IP-ERCC4ERCC1 complex in the incision process during ICL repair (64–66).

### 3.6 The role of ERCC4 in telomere maintenance

ERCC1–ERCC4 exhibits a genetic interaction with the telomere maintenance protein TRF2. Mice overexpressing TRF2 show hypersensitivity to ultraviolet radiation compared to normal mice, resulting in telomere loss in their chromosomes. Conversely, expression of ERCC1-ERCC4 in TRF2-deficient cells leads to increased chromosomal end fusion (67). Mechanistically, ERCC1-ERCC4 can cleave at the 3’ end of telomeres, causing telomere shortening and premature aging in mice. ERCC1-ERCC4 and TRF2 are associated through interaction with the SLX4 protein and collectively participate in regulating telomere length. The balance between ERCC1-ERCC4 and TRF2 expression may be crucial for expected lifespan and genome stability (68, 69).

## 4 Diseases and animal models associated with ERCC4 deletion

Previous studies have shown that ERCC4 gene defects can lead to genetic instability and carcinogenic effects. There have been numerous studies both domestically and abroad that have confirmed the association between ERCC4 gene polymorphisms and the occurrence of colorectal cancer, gastric cancer, lung cancer, breast cancer, glioma, and osteosarcoma (70, 71). Arora et al. demonstrated that lowering ERCC4 protein expression through shRNA sensitizes cells to DNA cross-linking agents (such as platinum derivatives), which are widely used in the treatment of breast cancer, prostate cancer, ovarian cancer, and other cancers (72, 73). Therefore, defects in the ERCC4 gene may lead to genetic instability and carcinogenic effects.

### 4.1 Xeroderma pigmentosum

XP is one of the most prominent diseases caused by NER defects and is a congenital condition that results in skin damage in sun-exposed areas of the body. XP patients also have an increased risk of developing lung and gastrointestinal cancers, suggesting a lack of NER defense against carcinogens from air and food. Additionally, approximately 20% of XP patients suffer from neurological disorders such as neurodegeneration, dementia, and microcephaly. Mutations in any NER-related enzyme genes (i.e., XPA, XPB, XPC, XPD, XPE, XPF, XPG) can cause XP. These XP patients exhibit clinical symptoms before the age of 2 and develop cancer before the age of 10. In contrast, patients with XPF (ERCC4) have milder symptoms, with later onset of skin cancer, possibly due to the fact that XPF mutations are usually point mutations occasionally resulting in frameshift, leading to the unstable truncation of the XPF protein (74, 75).

### 4.2 Cockayne syndrome

CS is a severe condition that causes accelerated aging. Common symptoms include neurodegeneration, intellectual disabilities, growth retardation, severe cataracts, retinal degeneration, microcephaly, vascular abnormalities, and pneumonia as a common cause of death. So far, mutations in the XPB, XPD, XPF, and XPG genes have been identified as occasional causes of CS. Due to defects in NER repair mechanisms, these mutations impede replication and transcription, leading to CS. Even a relatively slight level of unrepaired mutations within the transcribed genes is sufficient to significantly shorten lifespan (76, 77).

### 4.3 Fanconi anemia

FA is a disease characterized by progressive bone marrow failure and increased susceptibility to cancer. Currently, at least fifteen proteins have been identified to be associated with FA (78, 79). Previous studies involved whole-protein-coding gene sequencing of an FA patient, revealing mutations in the ERCC4 gene. Genetic, biochemical, and functional analyses of this mutation indicated a sharp decrease in the function of ERCC4 in interstrand crosslink repair (80). Furthermore, another case presented a combined phenotype of FA, XP, and CS symptoms. These findings highlight the multifunctional role of ERCC4 beyond DNA repair.

Currently, diseases associated with ERCC4 deficiency such as XP, CS, FA, etc., are closely linked to defects in the NER pathway. Therefore, repairing or compensating for NER pathway function may directly target the pathogenic mechanisms of these diseases. Effective treatment strategies are currently lacking, with symptomatic treatment being the mainstay (81). Research on causal treatments mainly involves stem cell therapy, gene therapy, and improving mitochondrial function, among others, which are still under further investigation (82). However, studies have shown that the combination therapy of PARP inhibitors and topoisomerase I toxins may be most effective against tumors lacking ERCC1-XPF. Platinum-based drugs like cisplatin, carboplatin, and nitrosoureas repair DNA damage induced by endogenous and environmental DNA-damaging agents through the NER pathway, thereby safeguarding the genome. Several clinical studies have demonstrated the efficacy of NER pathway-based treatments for diseases such as bladder cancer, soft tissue sarcoma, hepatoblastoma (83). However, NER pathway involvement encompasses multiple proteins and steps, and treatment involves complex molecular biology mechanisms, posing significant technical challenges that may require highly specialized medical professionals and laboratory conditions. Targeted therapy against this pathway may require precise regulation and substantial clinical trials to establish its safety and avoid unnecessary side effects (49, 84–86).

### 4.4 Construction of ERCC4 gene knockout mouse model

Previous research has mainly focused on the construction of ERCC1 gene knockout mice, with less emphasis on ERCC4 gene knockout mouse models. Tian M et al. generated XPF-mutant mice with a nonsense mutation designed in exon 23 to mimic human ERCC4 gene deletion. Homozygous pups had a weight that was 27% of the wild-type control or heterozygous mutants around 15 days after birth. Homozygous ERCC4 mutant mice died at approximately 3 weeks of age. Their organs appeared morphologically normal but noticeably smaller. However, hepatocytes from homozygous mutant mice contained enlarged nuclei. This severe phenotype resembles that of ERCC1-mutant mice, suggesting the role of ERCC1-ERCC4 as a complex (87). Janin Lehmann et al. successfully knocked out ERCC4 using CRISPR/Cas5 technology in fetal lung fibroblasts, which resulted in significantly increased sensitivity to cisplatin. Additionally, reduced NER and ICL repair capacity in these cells were assessed through genetic testing. Importantly, ERCC1 could not be detected in the nuclei of XPF-knockout cells, suggesting that a functional XPF/ERCC1 complex is necessary for ERCC1 to enter the nucleus (88).

## 5 ERCC4 as a potential regulator in IBD-related CAC development

The development of CAC may involve the formation of abnormal crypt foci, polyps, adenomas, and cancerous tissues (89–91). CAC initiation factors associated with IBD primarily involve repeated cycles of damage and healing in colonic epithelial cells (IECs). Factors contributing to CAC pathogenesis include the degree and duration of chronic inflammation, genetic susceptibility, and interactions with the microbiota. Over the past decade, significant progress has been made in understanding the molecular mechanisms underlying CAC development, thanks to the application of genomic, epigenomic methods, and genetically modified mouse models. However, the exact molecular mechanisms underlying the transition from inflammation to cancer remain unclear (13, 14, 23).

### 5.1 ERCC4 may be a potential regulatory factor for genetic mutations in IBD-related CAC

#### 5.1.1 p53 regulates the ERCC family and influences DNA repair

UC patients are more prone to develop colorectal cancer (13). It has been reported that persistent inflammation in the colon leads to increased cell numbers requiring repair due to intestinal barrier damage. Molecularly, telomere shortening, chromosomal instability, and p53 gene mutations also drive tumor development (92, 93). Telomeres are located at the ends of chromosomes and protect chromosome ends from DNA damage. Studies have found that telomere depletion plays two opposite roles in cancer: as a tumor suppressor mechanism when DNA damage checkpoints are intact, and as a mechanism for generating chromosomal instability when DNA damage checkpoint functions are impaired (54, 94, 95). Telomere length decreases with age in most human tissues, including the colon, and it has been hypothesized that short telomeres may partially explain the link between cancer and aging (96, 97). Previous studies have found genetic interactions between ERCC1–ERCC4 and the telomere maintenance protein TRF2. Mice overexpressing TRF2 are more sensitive to UV radiation, resulting in telomere loss. Conversely, ERCC1-XPF expression in TRF2-deficient cells leads to increased fusion of chromosome ends (98, 99). Previous studies have shown that UC patients have shortened telomeres in colon cells, particularly in those progressing to dysplasia and cancer. This hypothesis was supported by the observation of shorter telomeres in UC patients’ colonic epithelial biopsies compared to normal control groups (100, 101). Rosa et al. found that telomeres were shortest in low-grade dysplasia (LGD) samples from UC patients, longer in high-grade dysplasia (HGD) samples compared to LGD samples, showing the opposite result. P53 expression was lower in non-dysplastic biopsies but gradually increased in LGD and HGD (102). These results suggest a progression model of inflammation, telomere shortening, and high levels of DNA damage activation leading to colonic senescence in UC. Senescence acts as a tumor suppressor mechanism to prevent further development of colitis cells. Ultimately, some colonic cells bypass this mechanism and extend telomeres through activation of telomerase and loss of p53 function, resulting in progression to HGD and UC-related CAC (103).

**Figure 2:**
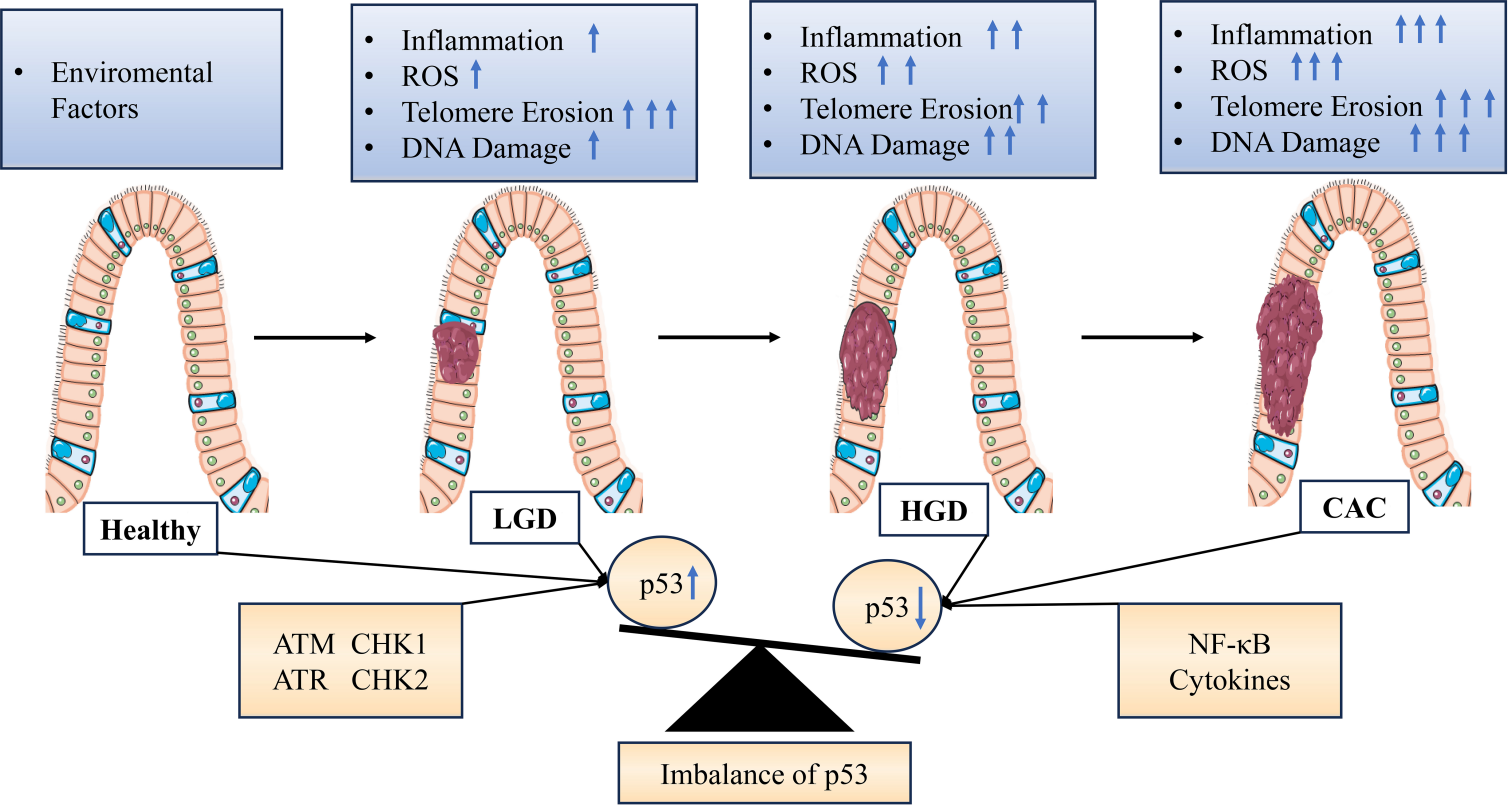
Graphical representation of stress signal-mediated dysregulation of p53 expression during progression from colitis to cancer. Various endogenous or exogenous stress signals are detected, which are transmitted by proteins in different signaling pathways within the cell leading to activation or inhibition of p53. Inflammation and aging are associated with decreased expression and/or activity of p53 in cells or tissues, resulting in impairment or inactivation of the p53 pathway and stress response. DNA damage is associated with increased expression and/or activity of p53 due to extensive ROS stimulation in the tissue. LGD, Low-grade dysplasia; HGD, High-grade dysplasia; ARF, Alternate reading frame; ATM, Ataxia telangiectasia mutant; ATR, Ataxia telangiectasia related; CHK, Checkpoint; ROS, Reactive oxygen species; NF-κB, Nuclear factor-κB.

Studies have shown that NER is tightly regulated by p53. In the case of NER, p53 affects DNA repair by activating downstream effectors such as gadd45a and p48-XPE. The observation of increased overall genomic repair with p53 supports the possibility of p53-dependent DNA repair *in vivo* (104). Recent studies have also found that p53 can regulate ERCC5 for NER (105). Watanabe et al. demonstrated that telomere shortening triggers DNA damage signaling, enhancing p53 transcriptional activity and inducing senescence or apoptosis, thereby inhibiting telomerase activity in IECS cells (106). Nancy et al. found downregulation of mRNA expression of telomere-binding proteins TRF1 and TRF2 in ulcerative colitis and Crohn’s disease, suggesting a role for these telomere-binding proteins in telomere regulation and possibly leading to telomere fusion and chromosomal abnormalities observed in UC (**Figure 2**) (107).

Although there is currently no specific mechanism-related study on the regulation of ERCC4 in the development of IBD-related CAC through p53, based on its role as a member of the XP family and its biological functions in DSB repair pathways and telomere maintenance pathways, it is hypothesized that ERCC4 may be a potential regulatory factor in the occurrence and development of ulcerative colitis and related cancers.

#### 5.1.2 ERCC4 may have potential value in inducing IBD intestinal epithelial damage and IBD-related CAC through the regulation of USP37 expression

It is known that USP37 is a member of the deubiquitinase family and can prevent the degradation of cancer proteins by deubiquitinating them in cancer. The first documented function of USP37 stems from Dixit and colleagues, who identified it in a proteomic screening where USP37 was observed to facilitate the interaction of the Anaphase Promoting Complex/Cyclosome (APC/C) with its associated proteins (108, 109). The deubiquitinase activity of USP37 is known to contribute to the timely onset of S-phase and progression through mitosis. Further analysis revealed that cells depleted of USP37 exhibit elevated levels of replication stress and DNA damage markers, including γH2A.X and 53BP1, particularly under conditions of replication perturbation. Depletion of USP37 also reduces cell proliferation and increases sensitivity to drugs inducing replication stress. Investigations into the heightened sensitivity revealed instability of checkpoint kinase 1 in the absence of USP37, thereby compromising its function (109, 110). Mechanistically, Chenming Wu et al. discovered that DNA double-strand breaks promote the phosphorylation of USP37 by ATM and enhance the binding between USP37 and the RecQ helicase BLM (111). Through bioinformatics analysis, we predicted that ERCC4 may regulate the expression of USP37.

**Figure 3:**
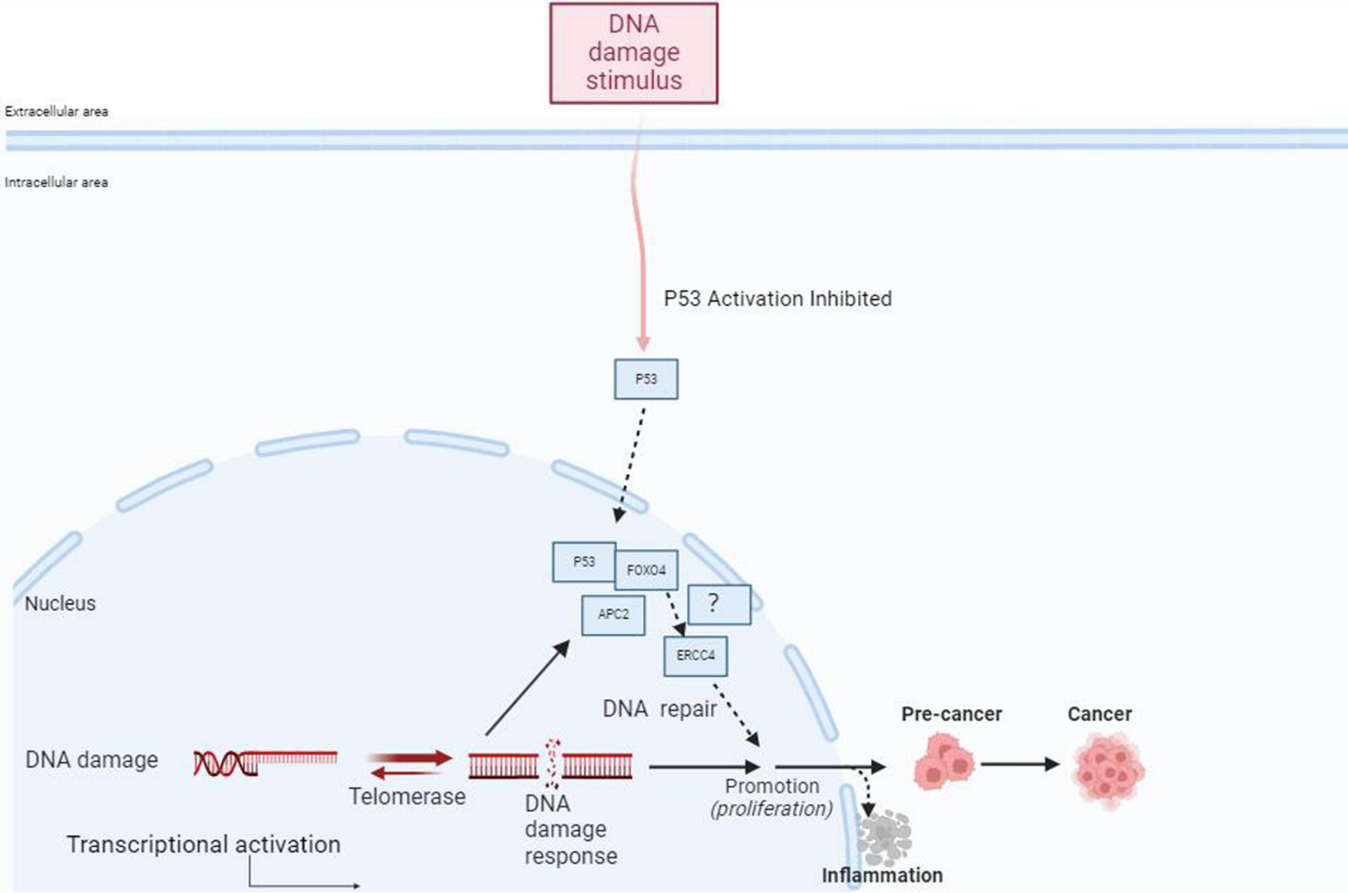
FOXO4 may be involved in the progression from IBD to inflammatory CRC through its mediation of ERCC4. External stimuli such as DNA damage can activate p53, which regulates the FOXO4 through APC2. FOXO4 may mediate the involvement of ERCC4 in the occurrence and progression of IBD to inflammatory CRC, participating in the process of DNA repair after cellular DNA damage. Additionally, FOXO4 can bind to p53 to regulate DNA repair after damage.

Previous reports have suggested that USP37 may participate in the process of DNA damage repair, but its specific role in nucleotide excision repair remains unclear. Through bioinformatics analysis, we predicted that ERCC4 may regulate the expression of USP37 (111–114). The interaction and mode of action between ERCC4 and USP37 in the development of IBD have not been reported. Our previous experiments revealed that the forkhead transcription factor FOXO4 downstream of the PI3K/AKT pathway can regulate the invasion and metastasis of colorectal cancer by participating in the APC2/β-catenin axis (115). Overexpression of FOXO4 (tumor suppressor) can reduce the expression of Snail (116). Snail is a zinc finger transcription factor that can inhibit the transcription of epithelial cell calcium adhesion protein (E-cadherin) and promote the process of epithelial-mesenchymal transition (EMT) (117, 118). Previous studies have also emphasized the role of USP37 in regulating Snail in EMT. USP37 plays an important role in promoting cancer cell migration, downregulating E-cadherin, and upregulating wave protein by stabilizing Snail expression (119–121). Wu et al.’s recent research reported the detailed mechanism of USP37-mediated SNAI1 deubiquitination in gastric cancer (GC) cells (**Figure 3**) (119). The specific role of the ERCC4-USP37-Snail pathway in the mucosal barrier injury and repair process induced by IBD inflammation is still unclear and requires further experimental confirmation.

### 5.2 ERCC4 may be involved in regulating chronic inflammation and immune microenvironment

#### 5.2.1 Oxidative stress damage

Previous studies have suggested that persistent intestinal chronic inflammation stimulation can lead to genetic mutations in intestinal epithelial cells, malignant cell proliferation, and the production of a large amount of activated immune cells generating ROS and reactive nitrogen intermediates, which can induce DNA damage or mutation. Inhibiting nitric oxide synthase activity can reduce adenoma formation and tumor occurrence (122, 123). Oxidative stress escalation is a hallmark of chronic inflammation, as activated cells of the innate immune system release a variety of reactive oxygen and nitrogen species (RONS) into the tissue microenvironment. These species include superoxide, hydrogen peroxide, hydroxyl radicals, and nitric oxide. RONS interact with cellular DNA, leading to diverse forms of DNA damage such as single and double-strand breaks, abasic sites, and nucleotide modifications. These damages contribute to tumorigenesis by influencing oncogenes or tumor suppressor genes. Thus, maintaining genomic integrity against ROS is essential for normal cellular function and the preservation of internal balance. in the realm of IBD, research underscores the association between elevated levels of RONS and disease activity, coupled with diminished antioxidant levels. Notably, heightened nitric oxide concentrations correlate with oxidative damage observed in both active and inactive IBD tissue samples (124, 125).

In human IBD, research indicates a correlation between heightened levels of RONS and disease activity, coupled with reduced antioxidant levels. For example, 8-oxo-7,8-dihydro-2’-deoxyguanosine, a base modification sensitive to oxidative stress, is frequently detected in inflamed and dysplastic tissues but is less prevalent in healthy mucosa (126). Likewise, increased concentrations of nitric oxide are associated with oxidative damage observed in both active and inactive IBD tissue samples. DDR are activated to address mutations triggered by reactive oxygen species, employing diverse repair mechanisms including NER (127). Moreover, NER modulates cell proliferation by instigating premature cellular senescence, an irreversible halting of the cell cycle process that curtails the amplification of defective DNA and the proliferation of mutant clones. Notably, in IBD, Sohn et al. observed augmented DDR (H2A.Xγ, phosphorylated checkpoint kinase 2) and aging (heterochromatin protein 1 γ) markers in inflamed tissue specimens from IBD patients (128, 129).

In addition, oxidative stress can also activate DNA nucleotide excision repair enzymes, including ERCC4. Stephen P et al. found that mice fed with azoxymethane (AOM) alone showed a significant increase in ERCC4 expression in intestinal epithelial cells after 5 weeks, suggesting that the DNA repair program is induced and maintained in colonic tissue in response to the DNA-damaging agent AOM-induced intestinal injury. However, they found that after inducing chronic colitis in mice using DSS or AOM/DSS multiple times, the expression of DDR genes MLH1, Anapc1, and ERCC4 mRNA significantly decreased, suggesting that the downregulation of ERCC4 expression after chronic colitis may be related to the occurrence of early tumors, which warrants further exploration (130). These results indicate that tissue-specific ERCC4 gene deletion triggers the gradual accumulation of persistent cytotoxic DNA damage.

#### 5.2.2 Immune cell-mediated immune response

During inflammation, the fate of cells depends on the relative balance between pro-tumor immune response and anti-tumor immune response. Like other solid tumors, colon inflammation-associated tumors are infiltrated by various types of immune cells. In other types of cancer, T cell infiltration is associated with poor prognosis. However, in the pathogenesis of CAC, immune surveillance leads to the detection and clearance of aberrant crypt foci, keeping the tumor in a dormant state (131, 132). Immune surveillance is crucial in the process of cancer cell metastasis, and the core role of cancer immune surveillance is attributed to cytotoxic CD8+ cells. After tumor-specific antigens are presented by dendritic cells (DC), CD8+ T cells are activated and release a large amount of cytotoxic cytokines, inducing tumor cell apoptosis (133, 134).

**Figure 4:**
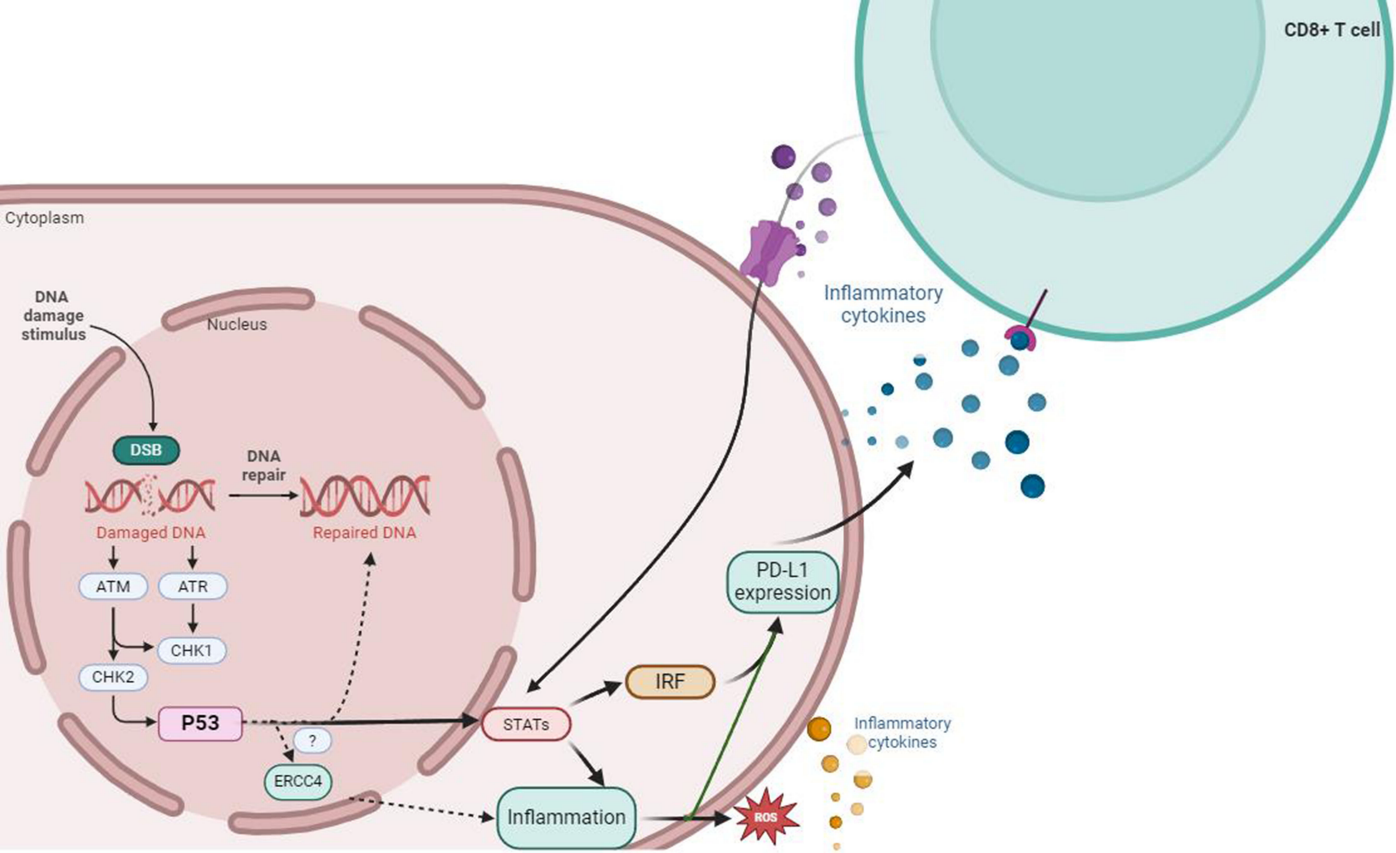
ERCC4 may serve as an important regulatory factor for the upregulation of PD-L1 expression induced by DNA damage signals and DDR defects. DNA damage and repair signals can induce the expression of PD-L1 mRNA, which depends on the activity of the ATM-ATR/CHK1 signaling pathway, while DDR defects may be associated with the upregulation of PD-L1 induced by DSBs. In the case of immune response, external stimuli such as DNA damage can activate the downstream STAT1/3-IRF through the ATM-ATR/CHK1 signaling pathway, which is crucial for the production of PD-L1 mRNA that can be activated at the transcriptional level. p53 may be involved in the process of DNA repair after cellular DNA damage through its mediation of ERCC4. Meanwhile, immune cells induced by inflammation (including large amounts of ROS from oxidative stress), such as CD8+ T cells, can secrete large amounts of pro-inflammatory cytokines and participate in the regulation pathway of PD-L1 by activating the ATM-ATR/CHK1 signaling pathway.

Programmed death 1 (PD-1) is an immune receptor expressed on activated CD4+ T cells, CD8+ T cells, and peripheral B cells. PD-1 inhibits T cell proliferation and interferon-gamma (IFN-γ) production. Programmed death ligand 1 (PD-L1) has been identified as the ligand for PD-1. The main function of PD-1/PD-L1 interaction is to regulate the autoimmune response in peripheral tissues (135, 136). It has been reported that human PD-1 gene or single nucleotide polymorphisms (SNPs) associated with enhanced PD-1 expression are related to human autoimmune diseases. Therefore, the interaction between PD-1 and PD-L1 is crucial for controlling the overall immune response in the human body (137, 138). PD-L1 is regulated by various inflammation-related transcription factors, including STAT1/3, interferon regulatory factor 1 (IRF-1), and nuclear factor kappa-B (NF-κB). Increasing evidence suggests that exogenous cellular stress induces upregulation of PD-L1 in cancer (139–142). IBD is a chronic inflammatory disease associated with DNA damage, and the DNA double-strand break repair pathway is involved in IBD-related dysplasia or CAC. Previous studies have found that mice with ERCC4 deficiency and mouse fibroblasts showed increased H2AXγ and chromosomal abnormalities such as radial structures, gaps, and breaks, which are manifestations of double-strand DNA breaks (DSB) after exposure to ionizing radiation (76). Naoya Ozawaet al. found that the expression of PD-L1 in UC and UC-related dysplasia/colorectal cancer tissues was significantly upregulated compared to sporadic colorectal cancer tissues and corresponding non-cancerous mucosa. In addition, the expression of PD-L1 in tumor-infiltrating CD8+ T lymphocytes in UC-related dysplasia/CAC tissues was higher than that in SCRC tissues (143). They also found that the upregulation of PD-L1 was associated with increased expression of γH2AX and IRF-1 in clinical UC-related dysplasia and colorectal cancer tissues, but not in SCRC tissues or corresponding non-cancerous UC mucosa. In cancer cells, PD-L1 is associated with the activation of DNA damage repair kinases such as ATR and ATM (**Figure 4**) (144, 145). Combining the biological role of ERCC4 in the DSB repair pathway, it can be hypothesized that ERCC4 affects the expression of PD-L1 on CD8+ T cells through the DSB/IRF-1 signaling axis in the intestinal tract of patients with UC and UC-related dysplasia/CAC, and may serve as a biomarker for inflammatory DNA damage in the future.

Macrophages have been proposed to play a promoting role in the development of IBD to tumors (146). M1 macrophages promote the tumor microenvironment by producing pro-tumor cytokines and have a proliferative effect on colon cells through the NF-κB and STAT3 pathways. Macrophage infiltration has also been observed in IBD-related CAC, and the level of macrophage infiltration is positively correlated with the degree of pathology (147, 148). Another study showed that the percentage of M2-like macrophages significantly increased in IBD-related cancer, indicating that macrophages polarize into anti-inflammatory or immunosuppressive phenotypes during the transition from dysplasia to cancer. Therefore, macrophages display both pro-inflammatory and anti-inflammatory mechanisms that contribute to IBD-related cancer (149–151). It can be imagined that M1 macrophages initially invade the inflamed intestinal mucosa in IBD and contribute to carcinogenesis by producing pro-inflammatory mediators. As inflammation becomes chronic, M1-like macrophages acquire the M2-like immune suppressive phenotype, which may further accelerate carcinogenesis. Further research is needed to better characterize the role of macrophages in IBD-related cancer.

Goulielmaki et al. conditionally depleted the expression of the ERCC1-ERCC4 complex in macrophages of mice, which led to Golgi dispersion, endoplasmic reticulum expansion, autophagy, and exosome release in infiltrated macrophages, causing metabolic abnormalities and increased glucose intake *in vivo*. Additionally, they observed macrophage infiltration in tissues, which promotes the release of cytokines such as IL6 and IL8, triggering chronic inflammation. Lymphocyte and monocyte aggregation was observed in the liver, lungs, and kidneys of mice, with elevated expression of cell adhesion molecules ICAM-1 and VCAM-1 (152). These findings suggest a potential role of ERCC4 in modulating macrophage function and influencing the progression of IBD-associated inflammation and carcinogenesis. However, the exact mechanisms and whether ERCC4 affects the process requires further investigation, considering its crucial role in the NER repair pathway.

#### 5.2.3 Cytokine-mediated immune response

During the development of CAC, intestinal epithelial cells (IECs) play two main roles. First, they utilize highly specialized cell types such as Paneth cells and goblet cells to prevent the invasion of intestinal pathogens. Disruption of the functional homeostasis of IECs leads to the infiltration and overactivation of innate immune cells. Additionally, IECs act as both producers and participants in cytokine and chemokine signaling, which affects the proliferation, migration, and survival of epithelial cells. This creates a favorable local environment for epithelial transformation and tumor development (153–155). The influx of immune cells into the intestinal epithelial layer and lamina propria forms a unique inflammatory microenvironment. Studies have shown that tumor-infiltrating monocytes, macrophages, and neutrophils secrete large amounts of pro-inflammatory cytokines such as IL-6, IL-8, and TNF-α, promoting tumor development in CAC models (155, 156). In a CAC mouse model, treatment with IL-6 neutralizing antibodies effectively inhibits chronic intestinal inflammation, while IL-6 knockout reduces tumor burden and significantly promotes IEC apoptosis (157–159). It has been found that XPF/XPG-mediated DNA damage can promote the expression of NF-κB target genes and host cell survival, while the expression of NF-κB target genes further promotes the secretion of inflammatory and cytokine factors such as TNF-α and IL-6, exacerbating the disease. Studies have shown the presence of high levels of IL-6, IL-8, and TNF-α in the serum of IBD patients. TNF-α, produced mainly by monocytes, macrophages, NK cells, and T lymphocytes, is an inflammatory cytokine that, upon binding to its receptor, activates NF-κB in cells through signal transduction. Activation of NF-κB upregulates the expression of interleukins IL-1 and IL-6, inducing inflammation and promoting cell survival, thus amplifying the inflammatory cascade reaction. This leads to the disruption of tight junctions and apoptosis of epithelial cells in the intestinal epithelium, impairing the intestinal mucosal barrier, and ultimately causing intestinal mucosal damage (160, 161). As mentioned earlier, patients with XP have a significantly higher risk of developing lung and gastrointestinal cancers, but the likelihood of developing IBD-associated CAC is still unknown. As a member of the XP family, it is currently unclear whether ERCC4 can affect the disease progression through the induction of cytokine secretion in the development of IBD and related CAC. Further research is needed to explore this relationship.

## 6 Conclusion

ERCC4 (XPF) and ERCC1 play important roles in genome maintenance as heterodimers involved in various DNA repair pathways and telomere maintenance. Due to the complexity of their many functions observed in mice and humans, it is difficult to individually elucidate the importance of each function. Further research is aimed at linking specific structural domains or amino acid sequences of the complex to certain functions. It is becoming increasingly clear that although ERCC4 is crucial for genome maintenance, elevated levels of ERCC4 mRNA and protein in cancer patients may paradoxically result in chemotherapy resistance and poor prognosis. The ERCC1/ERCC4 heterodimer thus becomes both a potentially interesting target for predicting treatment outcomes in patients and a potential target for drug therapy. The incidence of IBD has been increasing worldwide over the past 20 years. Importantly, IBD currently cannot be cured and is a lifelong disease, progressively worsening intestinal inflammation ultimately leading to structural and functional impairments in the digestive tract, seriously affecting patients’ growth, reproduction, as well as learning, working, and daily life. IBD, especially UC, is strongly associated with gastrointestinal tumors, particularly CRC, which can develop as colorectal cancer associated with IBD (CAC). Despite previous studies on the relationship between inflammation and tumors receiving significant attention from researchers worldwide, the mechanism by which ERCC4 influences the occurrence and development of IBD and subsequently leads to colorectal cancer at the protein level remains unclear. Further research is needed to understand how to control and prevent the occurrence and progression of inflammation-related colorectal tumors and reduce the incidence of inflammation-related colorectal tumors.

## Author contributions

RS: Investigation, Methodology, Writing – original draft. SW: Investigation, Methodology, Writing – original draft. YJ: Investigation, Writing – original draft. GZ: Investigation, Writing – original draft. ML: Methodology, Supervision, Writing – review & editing. YS: Methodology, Project administration, Supervision, Writing – review & editing.
